# A Case of Thrombotic Thrombocytopenic Purpura Possibly Induced by Graves’ Disease

**DOI:** 10.7759/cureus.29961

**Published:** 2022-10-05

**Authors:** Shifali Bains, Kriyesha Patel, Taranjit Bath, Pawanpreet Singh, Ravanjit Kaur, Parth Patel, Momal Jamali, Muhammad Abu Zar Ghaffari

**Affiliations:** 1 Internal Medicine, Punjab Institute of Medical Sciences, Jalandhar, IND; 2 Medicine, M.P. Shah Government Medical College, Jamnagar, IND; 3 General Medicine, Punjab Institute of Medical Sciences, Calgary, CAN; 4 Internal Medicine, Adesh Institute of Medical Sciences and Research, Bathinda, IND; 5 Medicine, Punjab Institute of Medical Sciences, Jalandhar, IND; 6 Medicine, Shri M.P. Shah Medical College, Jamnagar, IND; 7 Internal Medicine, Dow University of Health Sciences, Karachi, PAK; 8 Internal Medicine, Hayat Memorial Trust Hospital, Lahore, PAK

**Keywords:** case report, thrombotic thrombocytopenic purpura, ttp, graves' disease, thyrotoxicosis

## Abstract

Thrombotic thrombocytopenic purpura (TTP) has historically been diagnosed with a pentad of features, i.e., thrombocytopenia, micro-angiopathic hemolytic anemia (MAHA), fever, neurological abnormalities, and kidney failure. Traditionally, TTP cases have been described in healthy adults. However, their association with autoimmune diseases is now well documented in the literature. There is limited availability of literature on the association between TTP and Graves' disease (GD). Here, we report a case of an adult female, a known case of Graves’ disease, who has now been diagnosed with an acquired case of TTP. The presence of MAHA associated with thrombocytopenia was considered a clinical diagnosis of TTP and the patient immediately underwent plasma exchange (PEX), which led to the resolution of complaints. Hyperthyroidism cases should be adequately followed up as clinical severity could lead to the emergence of TTP.

## Introduction

Thrombotic thrombocytopenic purpura (TTP) is a primary thrombotic micro-angiopathy (TMA) caused by persistently reduced function of a metalloprotease that cleaves the von Willebrand factor (vWF); ADAMTS13 (a disintegrin and metalloprotease with a thrombospondin type 1 motif, member 13), causing small vessel platelet-rich thrombi, thrombocytopenia, and microangiopathic hemolytic anemia [1]. Most cases are acquired due to ADAMTS-13-specific autoantibodies. In a nutshell, the altered multimers of vWF then form, which causes to produce platelet thrombi in the microcirculation, leading to hypoxemia, thrombocytopenia, and red blood cell rupture (called fission). TTP has a high mortality rate [2].

In known cases of Graves' disease, immune thrombocytopenic purpura (ITP) and pernicious anemia should be assumed and ruled out first. However, the presence of micro-angiopathic hemolytic anemia (MAHA) associated with thrombocytopenia is considered a clinical diagnosis of TTP. The association, particularly the causal relationship, between Graves' disease (GD) and TTP has not been well studied [3]. Plasma exchange (PEX) and glucocorticoids are mainstay management strategies. Here, we describe the case of a 41-year-old Asian woman who has a history of hyperthyroidism and is now presenting with newly developed features of TTP.

## Case presentation

A 43-year-old Asian female patient presented to the emergency department of a tertiary care hospital with complaints of dyspnea, weakness, and dizziness. Her symptoms started a day earlier and have since worsened. She was also having occasional palpitations and losing sleep. She has been a known case of hyperthyroidism for nearly two years now and is being treated with antithyroid drugs, methimazole, and occasionally propranolol. However, she admitted to her recent lack of compliance with the medications.

A physical examination revealed a normal body temperature, 20 breaths/min, and 107 pulse/min. She was alert and anxious, and her thyroid was enlarged on palpation. The rest of her physicals, including her neurological and dermatological examinations, were normal. Preliminary laboratory tests revealed that her hemoglobin was 7.6 g/dL (normal range 12-16 g/dL), and her thrombocytes were 14 × 103/μL (150-450 × 103/μL). Her peripheral blood smear showed numerous schistocytes, her reticulocyte count was 2.9% (0.4-2.5%), her bilirubin was 2.8 mg/dl (0.1-1.0 mg/dl), and her lactate dehydrogenase was increased by 320 IU/L (91-200 IU/L). However, her electrolytes, renal function tests, fibrinogen, and coagulation levels were all within normal ranges. She was also evaluated for the presence of end-organ damage. Her ECG and serum troponin-I were normal. An abdominal ultrasound examination was unremarkable. Radiological investigations of the brain, however, were not ordered in the scenario of the absence of any neurological findings on repeated physical examinations.

On the basis of history, physical examination, and initial investigations, systemic conditions that produce MAHA and thrombocytopenia were ruled out. The differential diagnoses of primary TMAs were considered. However, given the absence of significant renal impairment and a narrow spectrum of clinical presentation, a presumptive diagnosis of TTP was made. She had a PLASMIC score [4] of 8 and was considered a "high probability of TTP" (Table 1).

**Table 1:** Calculating the PLASMIC score for estimating the likelihood of severe ADAMTS13 deficiency in adults with suspected TTP [4] PLASMIC: platelet count; combined hemolysis variable; absence of active cancer; absence of stem-cell or solid-organ transplant; MCV; INR; creatinine; ADAMTS13: a disintegrin and metalloprotease with a thrombospondin type 1 motif, member 13; TTP: thrombotic thrombocytopenic purpura

PLASMIC Score Calculator			Present	Absent
1	Platelet count <30,000/μL [<30 × 10^9^/L]		1	0
2	One or more indicators of hemolysis			
	(i)	Reticulocyte count (percentage) >2.5%	1	0
	(ii)	Haptoglobin undetectable	1	0
	(iii)	Indirect bilirubin >2.0 mg/dL [>34 μmol/L]	1	0
3	No active cancer in the preceding year		1	0
4	No history of solid organ or hematopoietic stem cell transplant		1	0
5	Mean corpuscular volume <90 femtoliters		1	0
6	International normalized ratio <1.5		1	0
7	Creatinine <2.0 mg/dL [<177 µmol/L]		1	0
Interpretation				
PLASMIC score (points)			Risk of severe ADAMTS13 deficiency	
0–4			Low risk	
5			Intermediate risk	
6–9			High risk	

PEX and glucocorticoids were commenced immediately. Before initiating the treatment, her ADAMTS 13 activity level was sent.

Further evaluation revealed that her TSH (thyroid-stimulating hormone) was suppressed markedly and that her free T4 was highly elevated. A thyroid ultrasound indicated increased perfusion. On the basis of her ultrasound findings, she was diagnosed with Graves' disease, and during this time her methimazole dose was increased to 20 mg, and she was started on propranolol 20 mg twice daily. A few days later, her ADMTS 13 activity returned and was significantly below 10% (normal range 50-160%). She underwent multiple PEX sessions during which her condition improved noticeably on clinical and laboratory examination and eventually underwent a total thyroidectomy.

## Discussion

Thrombotic thrombocytopenic purpura is a very rare disorder, with nearly three cases per million occurring in a year [5], and is three times more commonly found in women than in men [6,7]. It usually encompasses microangiopathic hemolytic anemia, thrombocytopenia, and renal injury [5].

TMA is marked by the formation of extensive thrombi in the smaller vessels, involving more than one organ [6]. The existence of the typical pentad is no longer essential for making a clinical diagnosis of TTP. The renal and neurological findings show signs of damage later in the course of the disease; however, the arrival of PEX has successfully reduced the progression of TTP to these classic findings of pentad [6,7]. Current guidelines suggest that the coincidence of platelet deficiency and MAHA after ruling out other causes is sufficient for making a clinical diagnosis of thrombotic thrombocytopenic purpura. The diagnosis is, however, confirmed when less than 10% activity of ADAMTS13 is found in the plasma [8].

Patients with autoimmune disorders are at risk of developing additional autoimmune diseases [9]. The correlation of TTP with Graves' disease is now well documented in the literature [10]. Four such cases have already been reported [2,11-13].

The confirmatory status of Graves' disease as an inciting factor of TTP has yet to be fully established. Nevertheless, elevated levels of autoantibodies against ADAMTS13 and endothelial activation have been found in cases of Graves' disease. The theory is also supported by the repletion of ADAMTS13 activity with the management of Graves' disease, even without PEX therapy, in certain cases [2,10].

Management is based on the risk stratification of each patient under the pretext of clinical and laboratory evaluation present at the time of diagnosis [14]. A presumptive diagnosis is an indication to initiate therapeutic PEX, glucocorticoids, rituximab, and, in selected patients, caplacizumab. This potentially life-saving management could not be delayed while awaiting the confirmatory test, i.e., the ADAMTS 13 activity level [5]. PEX, once a day, is considered the treatment of choice. It causes the removal of antibodies and restores ADAMTS13 activity to its normal levels. If PEX cannot be made available, fresh frozen plasma transfusion should be considered. Thrombocyte transfusion is not indicated unless there is a risk of fatal bleeding [14].

## Conclusions

Primary thrombotic microangiopathy in a previously known case of hyperthyroidism should raise the suspicion of immune TTP. The classic pentad of TTP is rarely observed these days due to the availability of PEX. Henceforth, MAHA and hemolytic anemia coincidentally presenting in a patient could be considered sufficient for making a clinical diagnosis and initiating the management plan, particularly when an association between TTP and hyperthyroidism disease has already been established. Regular follow-ups of patients with hyperthyroidism and consistent compliance with the treatment regimen could be considered the prime preventive strategies, which, however, are yet to be fully established.
